# Effects of mental fatigue on biomechanical characteristics and risk associated with non-contact anterior cruciate ligament injuries during landing

**DOI:** 10.3389/fbioe.2025.1582873

**Published:** 2025-05-27

**Authors:** Bosong Zheng, Zeyang Zhang, Zeyi Zhang, Youping Sun, Yao Xiao, Mengjie Li

**Affiliations:** ^1^ College of Physical Education and Health, East China Normal University, Shanghai, China; ^2^ Key Laboratory of Adolescent Health Assessment and Exercise Intervention of Ministry of Education, East China Normal University, Shanghai, China; ^3^ College of Physical Education, Chengdu Sports University, Chengdu, China

**Keywords:** mental fatigue, stop-jump, single-leg landing, non-contact anterior cruciate ligament injury, sports biomechanics

## Abstract

**Objective:**

To investigate and compare the effects of mental fatigue (MF) on biomechanical characteristics associated with non-contact anterior cruciate ligament injury (NC-ACLI) in male college students during stop-jump (SJ) and single-leg landing (SL), and whether it increases NC-ACLI risk.

**Methods:**

MF was induced by a 45-min Stroop task and measured using the visual analogue scale for MF (VAS-MF), while motion capture, force platforms, and surface electromyography (SEMG) evaluated biomechanical variables before and after MF induction in thirty-six subjects. Kinematic, kinetic, and SEMG data were analyzed using two-factor repeated measures ANOVA and rank-based nonparametric ANOVA.

**Results:**

Following MF induction, VAS-MF scores increased significantly. The ANOVA showed that in both maneuvers, peak vertical ground reaction force increased, while ankle dorsiflexion angle and knee flexion moment decreased. In SJ, knee flexion and internal rotation angles and internal rotation moment decreased, whereas knee abduction moment increased; these parameters did not change significantly in SL. The median frequency of biceps femoris SEMG decreased in SL but remained unchanged in SJ. No significant differences were found in hip flexion angle, knee adduction angle, or SEMG measures of rectus femoris, tibialis anterior, gastrocnemius lateral head, or biceps femoris root mean square.

**Conclusion:**

MF partly influences NC-ACLI biomechanics and increases risk in both maneuvers—more pronounced in SJ than in SL—potentially due to MF’s impact on central nervous system function, cognition, and attention. MF should be considered in NC-ACLI prevention strategies.

## 1 Introduction

Anterior cruciate ligament (ACL) injury is among the most common, severe and rapidly escalating knee injuries in sports over the past two decades (Maniar et al., 2022b). In Australia, it exhibits annual growth rates of 10.4% and 7.3% among adolescent females and males respectively (Maniar et al., 2022b), while in the United States, the incidence stands at 68.6 per 100,000 person-years (Sanders et al., 2016). It is estimated that 71.1% of all ACL injuries are non-contact (NC-ACLI) (Motififard et al., 2024), predominantly occurring during single-leg landing (SL) and stop-jump (SJ) maneuvers in sports such as soccer, basketball, and volleyball (Lewis et al., 2018; Chia et al., 2022; Childers et al., 2024; Crotti et al., 2024). Beyond diminishing athletic performance (Ardern et al., 2014), ACL injuries trigger joint instability, chronic pain and early osteoarthritis, thereby jeopardizing both the physical and mental wellbeing of athletes (Khan et al., 2019; Selin et al., 2024). Although reconstructive surgery can restore function, it is financially burdensome—with an average lifetime cost of $38,121 (Mather et al., 2013)—and most patients struggle to regain pre-injury activity levels while remaining at heightened risk for secondary injuries (Selin et al., 2024).

Early studies have demonstrated that NC-ACLI typically occurs within approximately 40 ms after SL and SJ maneuvers (i.e., from initial plantar contact to peak vertical ground reaction force (vGRF)) (Krosshaug et al., 2007; Koga et al., 2018), leading to the formulation of four classical injury explanatory theories—ligament dominance, quadriceps dominance, trunk dominance, and leg dominance (Boden et al., 2010; Hewett et al., 2010). Based on the aforementioned theories, subsequent research has confirmed that axial compressive loads exceeding the ACL tissue damage threshold represent a key injury mechanism (Boden and Sheehan, 2022), and that this mechanism is modulated by a constellation of biomechanical variables across the sagittal, frontal, and horizontal planes (Boden and Sheehan, 2022; Beaulieu et al., 2023). Specifically, higher vGRF directly increases axial compression in the lower extremities (Devita and Skelly, 1992; McNitt-Gray, 1993), thereby elevating the risk of ACL injury. In the sagittal plane, reduced knee, hip, and ankle flexion angles compromise the lower extremities’s capacity to absorb impact forces, further intensifying axial loads on the ACL and indirectly heightening injury risk (Schmitz and Shultz, 2010; Koga et al., 2018; Markolf et al., 2018; Bates et al., 2019; Boden and Sheehan, 2022). In the frontal and horizontal planes, excessive knee abduction and internal rotation angles, along with their associated moments, increase ACL stress and decrease its injury threshold (Kiapour et al., 2016; Bates et al., 2017; Boden and Sheehan, 2022; Schweizer et al., 2022). Moreover, quadriceps dominance, indicative of an imbalance between quadriceps and hamstring strength, further reduces the ACL injury threshold by increasing knee extension moments in the sagittal plane (Boden and Sheehan, 2022). Additionally, inadequate trunk control amplifies force line excursions in the frontal or horizontal plane, further elevating injury risk (Chou et al., 2023). Hence, identifying the risk factors that precipitate abnormalities in these biomechanical variables is crucial for preventing NC-ACLI and mitigating the risk of re-injury.

In recent years, fatigue has emerged as a potential intrinsic factor driving NC-ACLI–related biomechanical abnormalities (Carter and Micheli, 2011; Pfeifer et al., 2018), yet macroscopic evidence remains conflicting. Bourne et al. (2019)noted that the relationship between fatigue—as inferred from match playing time or external load—and NC-ACLI incidence is highly heterogeneous: Yeomans et al. (2021) reported that 83% of NC-ACLIs occur in the fourth quarter, whereas Della Villa et al. (2020) found injuries more frequent early in play. Such discrepancies suggest that equating fatigue solely with external load (e.g., distance run, duration of play) fails to capture ACL injury mechanisms fully (Benjaminse et al., 2019). In sports biomechanics, fatigue is typically divided into physical/physiological and mental/psychological (MF) categories (Tanaka et al., 2011; Pageaux and Lepers, 2018). Findings on physical fatigue’s impact on NC-ACLI risk are inconsistent. Declines in muscle strength, proprioception, and neuromuscular coordination following high-intensity or prolonged exercise (Zago et al., 2021; Song et al., 2024) may reduce hip and knee flexion angles while increasing GRF and knee abduction at landing, thereby elevating injury risk (Alentorn-Geli et al., 2009; Hodel et al., 2024). Conversely, fatigue-induced slowing of movement can increase hip and knee flexion and reduce GRF—a “spontaneous protection” strategy (Bourne et al., 2019). These contrasting effects indicate that physical fatigue alone cannot fully explain NC-ACLI risk.

In contrast, MF is a psychophysiological state triggered by prolonged, high-intensity cognitive activity or mental load (Van Cutsem et al., 2017; Pageaux and Lepers, 2018), fundamentally tied to “cognitive overload accumulation.” It commonly impairs endurance (Marcora et al., 2009; Lopes et al., 2020), balance (Hachard et al., 2020; Qu et al., 2020; Varas-Diaz et al., 2020), decision-making (Fortes et al., 2019; Fortes et al., 2020; Gantois et al., 2020), responsiveness (Englert and Bertrams, 2014; Smith et al., 2016a; Van Cutsem et al., 2019), and sensitivity and accuracy (Badin et al., 2016; Smith et al., 2017; Le Mansec et al., 2018; Filipas et al., 2021), thereby undermining athletic performance. Although MF frequently co-occurs with physical fatigue during competition and high-intensity training in sports where ACL injuries are common (e.g., basketball, soccer, volleyball) (Russell et al., 2019a; Russell et al., 2019b; Russell et al., 2022), numerous studies indicate that MF can be harmful independently of physical fatigue (Lew and Qu, 2014; Behrens et al., 2018; Kong et al., 2023). Schampheleer and Roelands (2024) further reported that MF may compromise the efficient regulation of joint movement patterns during landing or jumping, thereby exacerbating abnormal knee loads and moments and elevating ACL injury risk. However, there remains a dearth of empirical studies directly examining the independent effects and underlying mechanisms of MF on the biomechanical characteristics and risks associated with NC-ACLI during landing maneuvers.

A few studies provide indirect support. For instance, MF has been found to increase the likelihood of slip initiation and impair the ability to detect and respond to slips (Lew and Qu, 2014). In older adults, it increases gait variability during dual-task walking, elevating the risk of falls (Behrens et al., 2018). Similarly, in individuals with functional ankle instability, MF reduces ankle stiffness during unintended side-cutting maneuvers, increases ankle inversion and knee valgus angles, and potentially raises the risk of ankle re-injury (Kong et al., 2023). From a cognitive-neuroscience perspective, MF can cause glutamate accumulation in the lateral prefrontal cortex, which disrupts synaptic transmission and impairs cognitive processes, thereby reducing an individual’s ability to cope with cognitive loads (Proost et al., 2022; Wiehler et al., 2022). In addition, a recent review highlighted that increased cognitive load alters lower limb biomechanical characteristics associated with NC-ACLI during landing (Jiménez-Martínez et al., 2024). This suggests that MF may exacerbate abnormal landing patterns and heighten NC-ACLI risk through similar cognitive-neuromuscular regulatory mechanisms. However, this hypothesis requires further empirical validation.

Therefore, the present study primarily examined whether MF influences NC-ACLI risk by comparing key biomechanical variables in male college students performing SJ and SL maneuvers before (pre-MF) and after (post-MF) the induction of MF. Additionally, we provided a preliminary discussion of possible underlying mechanisms, drawing on existing literature to further our understanding of how psychological and psychiatric factors may contribute to NC-ACLI risk. The findings are expected to provide a theoretical basis for refining ACL injury risk assessment models and guiding the development of targeted preventive strategies, while also offering additional support for further clarifying the role of psychological/psychiatric factors in sports injury risk. Building on the above literature and reasoning (Kong et al., 2023; Jiménez-Martínez et al., 2024; Schampheleer and Roelands, 2024), we hypothesized that, relative to pre-MF, the post-MF state would yield higher vGRF, larger knee abduction/internal-rotation angles and moments, and smaller hip, knee, and ankle flexion angles during SJ and SL landings, thereby increasing NC-ACLI risk. We further hypothesized that MF-induced biomechanical changes would be task-dependent, with the inherently more complex SJ maneuver experiencing greater adverse effects compared to SL. All abbreviations are listed in Table 1.

**TABLE 1:** Abbreviations.

Abbreviation	Definition
ACL	Anterior cruciate ligament
CNS	Central nervous system
GRF	Ground reaction force
MF	Mental fatigue
MDF	Median frequency
NC-ACLI	Noncontact anterior cruciate ligament injury
RMS	Root mean square
SEMG	Surface electromyography
SJ	Stop-jump
SL	Single-leg landing
vGRF	Vertical ground reaction force

## 2 Methods

### 2.1 Participants

Prior to open recruitment, the sample size was estimated using G*Power 3.1 software with the “ANOVA: Repeated measures, within factors” option selected under “F tests.” Based on results from previous studies (Smith et al., 2016b; Greco et al., 2017; Fortes et al., 2020), the following parameters were set: effect size (f) = 0.25, significance level (α) = 0.05, test power (1-β) = 0.95, number of groups = 1, number of measurements = 4, correlation among repeated measures = 0.5, and non-sphericity correction = 1. The calculation determined a minimum required sample size of 36. To account for potential attrition, 39 subjects were recruited. Recruitment was conducted at East China Normal University. Recruitment criteria included: 1) males aged 18–25 years (Smith et al., 2016a; Fortes et al., 2020); 2) regular participation in ball games (e.g., basketball, soccer, volleyball) at least 3 times/week for ≥1 h/session; 3) no congenital deformities of the foot, ankle, knee, pelvis, or spine; 4) no previous lower limb surgery; 5) no severe head or lower extremity injuries in the past year; 6) no acute lower extremity injuries in the past 3 months; 7) no color blindness or color vision deficiency; 8) no cardiopulmonary diseases, sleep disorders, or psychological/psychiatric conditions (Pageaux et al., 2013). The experimental protocol was approved by the Medical Ethics Committee of East China Normal University (Approval No. 2024; No. 31), and all participants provided informed consent, which was filed in advance in the China Medical Research Filing Information System (Filing No. HSR-24–000,654).

### 2.2 Protocol

To minimize the confounding effects of individual differences and accurately reflect the task-dependent effects of MF on NC-ACLI-related biomechanical characteristics, a within-subjects two-factor repeated-measures design (maneuver: SJ vs SL; mental state: pre-MF vs post-MF) was adopted, requiring all participants to complete MF induction and corresponding tests in a randomized order. SJ and SL maneuvers were specifically selected because: 1) both are recognized as high-risk movements associated with NC-ACLI (Lewis et al., 2018; Chia et al., 2022; Crotti et al., 2024); and 2) inherent biomechanical and neuromuscular complexity differences between these two maneuvers may clarify whether MF disproportionately exacerbates biomechanical risk factors during the more challenging SJ compared to the relatively simpler SL task (Peebles et al., 2020). Participants completed a familiarization phase and a formal phase at the same time points, separated by at least 48 h.

**FIGURE 1:**
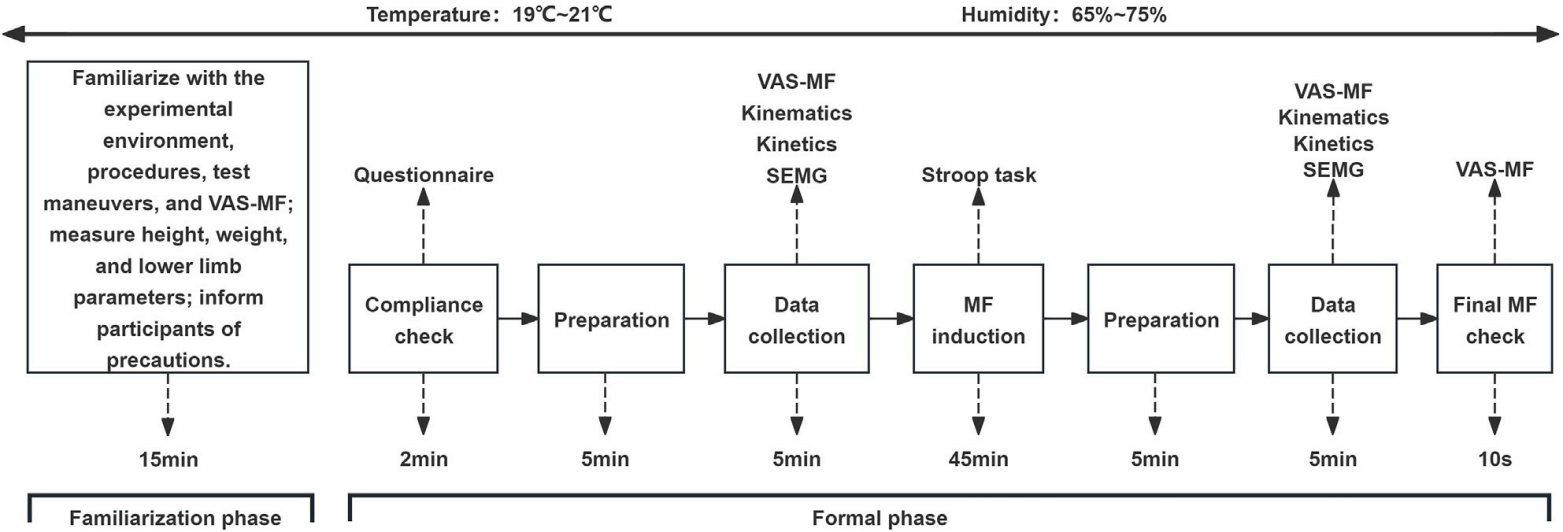
Experimental flowchart.

During the familiarization phase, participants were introduced to the experimental environment, the procedures (Figure 1), the test maneuvers (see 2.4), the induction task, and the use of the visual analogue scale for mental fatigue (VAS-MF) (see 2.3). Physical parameters were measured (see 2.4), and participants practiced the intervention task and follow-up tests for at least 5 min to ensure proficiency. Additionally, participants were instructed to maintain adequate sleep (≥8 h/day) 48 h before the experiment (Filipas et al., 2019; Thompson et al., 2019) and to avoid stimulants such as alcohol, nicotine, caffeine, and high-intensity physical activity (Azevedo et al., 2016; Franco-Alvarenga et al., 2019). Participants were also instructed to avoid tasks involving high cognitive loads (e.g., prolonged screen use) on the day of the experiment.

During the formal phase, compliance with these requirements was first verified through a questionnaire. Participants then wore standardized sneakers and leggings, performed a 3-min jogging warm-up, and practiced the test maneuvers until they were fully proficient. After the warm-up, three researchers worked together to attach reflective markers and surface electromyography (SEMG) testers to the participants. Baseline MF levels were assessed, and pre-test maneuver data were collected in a designated area. Participants then performed a 45-min Stroop task under the guidance of a trained researcher. Following the Stroop task, the same warm-up and testing procedures were conducted as in the pre-test to assess MF levels and collect post-test maneuver data. At the end of the post-test, the MF levels were reassessed. The total time from the completion of the Stroop task to the end of maneuver data acquisition was approximately 10 min, falling within the effective influence range of MF (Veness et al., 2017; Smith et al., 2019). The laboratory environment was controlled at 20°C ± 1°C and 70% ± 5% humidity during both visits.

### 2.3 MF induction and evaluation

#### 2.3.1 Induction

Referring to previous studies (Badin et al., 2016; Lopes et al., 2020), a 45-min Stroop task was used to induce MF. The procedure was as follows: On a 16-inch computer screen (ASUS UX3402Z, China), the Chinese characters for “red,” “green,” “blue,” and “yellow” were randomly presented, with each character displayed in one of these colors. In 50% of the trials, the color did not match the meaning of the character. Participants were instructed to respond to the color of the character by pressing a key. Each character appeared for 1 s, followed by a 1-s blank screen before the next character appeared. The task consisted of 1,350 judgments. Incorrect responses or delays of more than 1.5 s were met with an audible beep. Before starting, participants were instructed to maintain focus and informed that their performance (accuracy and speed) would determine their reward. The task was conducted in a quiet, isolated room, supervised by a staff member, and run using E-prime 3.0 software (Psychology Software Tools, Pittsburgh, PA, United States).

#### 2.3.2 Evaluation

Existing studies have used significant differences in VAS-MF scores before and after MF induction as a criterion for successful induction and have demonstrated high sensitivity and utility (Pageaux et al., 2015; Smith et al., 2019), outperforming physiological and cognitive metrics, such as EEG, heart rate variability, response time, and response accuracy (Penna et al., 2018; Smith et al., 2019; Filipas et al., 2021). Therefore, the present study also employed VAS-MF to assess MF severity. Subjects were asked to rate their fatigue on a 100-mm straight line, from “no fatigue” (0) to “complete fatigue” (100). To address potential ambiguity in MF perception among college students, a clear and uniform definition of MF was provided during the familiarization session, and standardized instructions were given to ensure they accurately understood and evaluated their fatigue levels, minimizing bias in self-reports.

### 2.4 Data collection

#### 2.4.1 Instrumentation and setup

**FIGURE 2:**
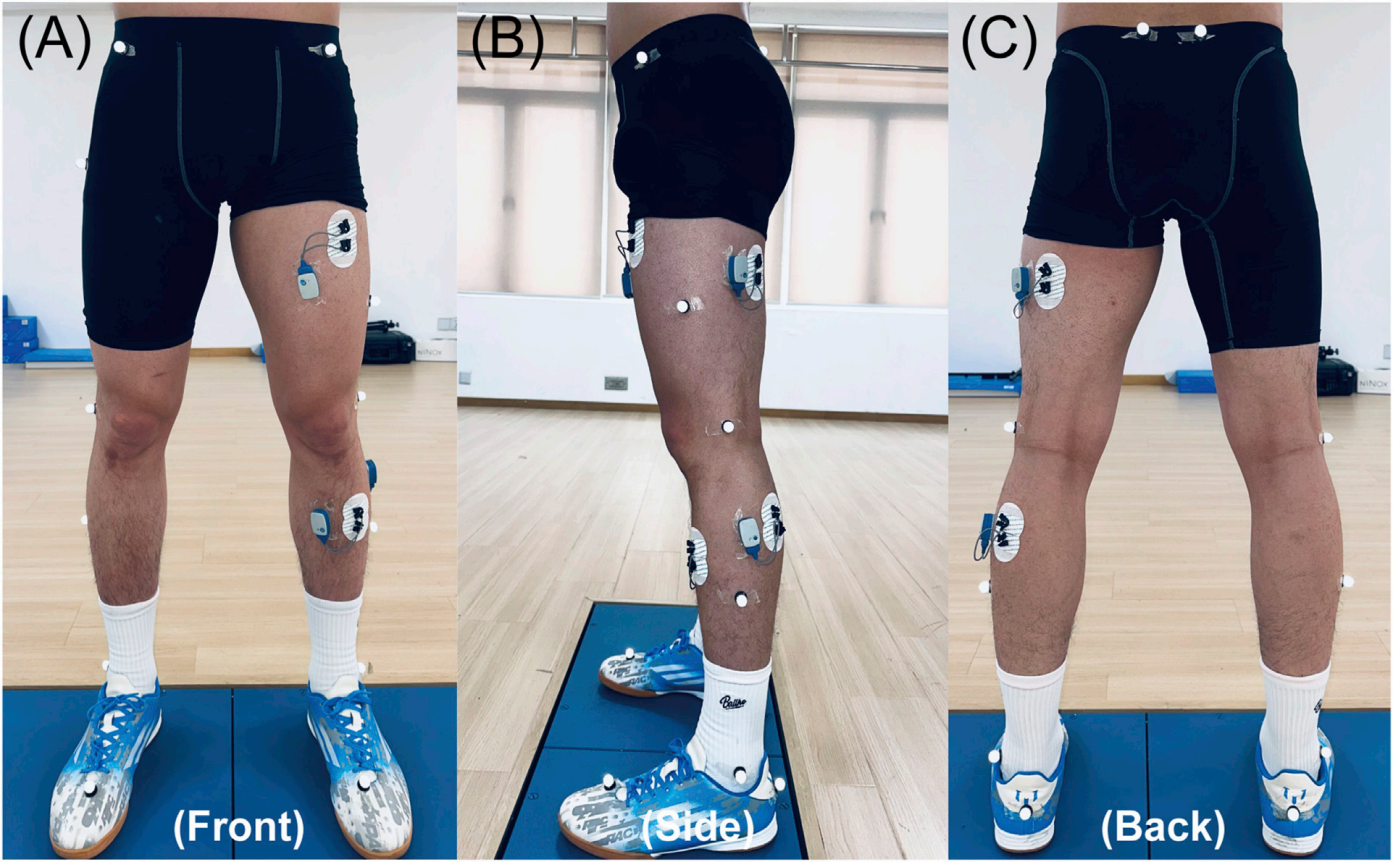
Lower-limb marker and SEMG electrode setup. **(A–C)** Front, side, and back views, respectively.

In this study, the Plug-in Lower Body Ai model was used to measure parameters such as height, weight, lower limb length (distance from the anterior superior iliac spine to the medial ankle), knee width (medial-lateral knee width), and ankle width (spacing between the medial and lateral ankles), following the Vicon system’s instructions. Reflective marker dots were placed on 16 body locations (left/right anterior superior iliac spine, left/right anterior thigh, left/right lateral femoral condyles, left/right anterior tibialis, left/right posterior superior iliac spine, left/right external ankle, left/right heel, and left/right ball of the foot). According to SEMG for the Non-Invasive Assessment of Muscles guidelines (Hermens et al., 1999), electrodes were placed on the preferred stance leg at the muscle bellies of the rectus femoris, long head of the biceps femoris, gastrocnemius lateral head, and tibialis anterior, aligned with the direction of muscle fibers (Figure 2). A 200 Hz 12-camera infrared high-speed motion capture system (Vicon V2.2, Vicon Motion Systems Ltd., Oxford, United Kingdom), 1,000 Hz 3D force plates (AMTI BMS400600, AMTI, Watertown, MA, United States), and 2000 Hz SEMG system (Ultium EMG, Noraxon United States Inc., Scottsdale, AZ, United States) were used to synchronously collect 3D coordinates, GRF, and SEMG data.

#### 2.4.2 Testing procedures

**FIGURE 3:**
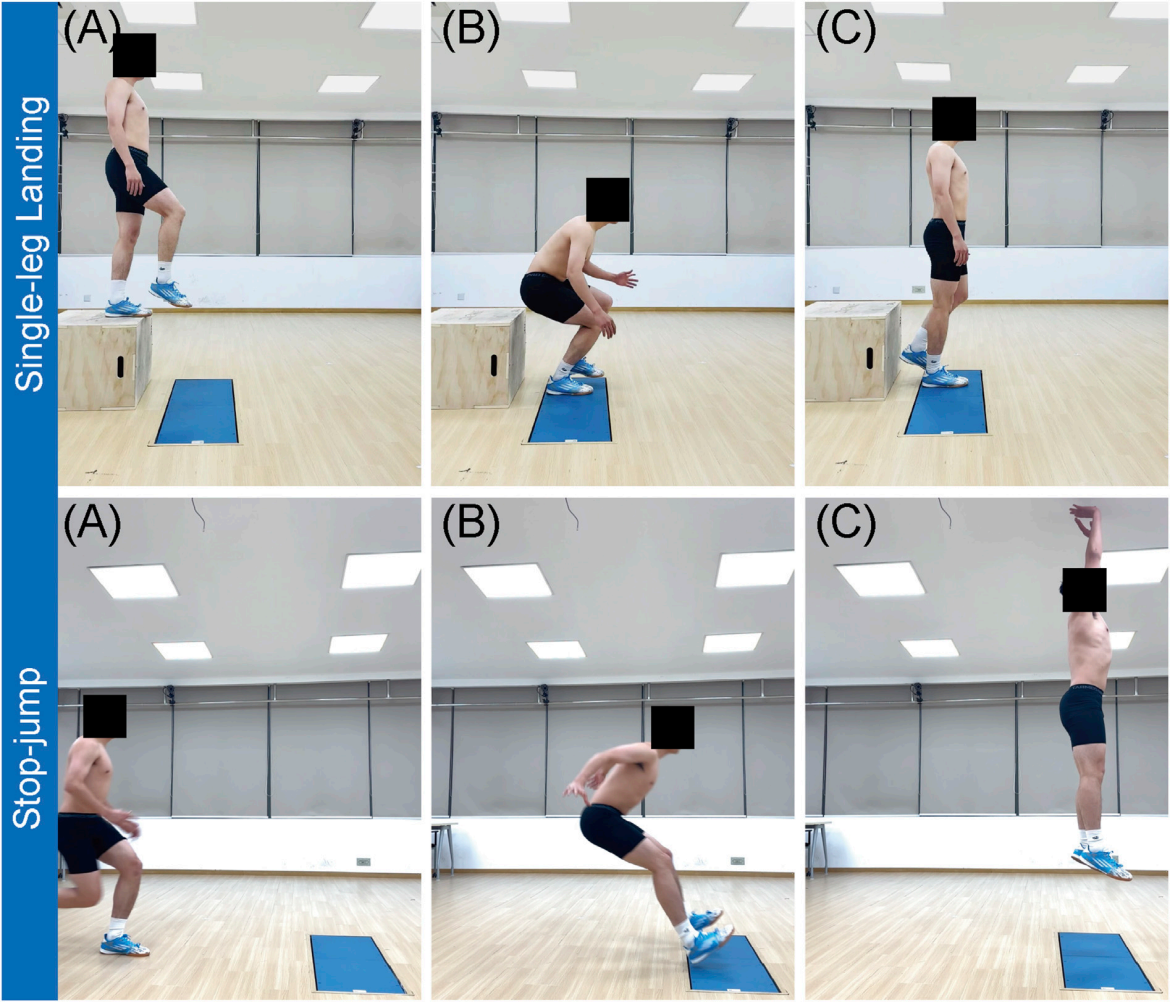
Schematic of test maneuvers. Top row **(A–C)** shows the sequence of the single-leg landing from start to finish. Bottom row **(A–C)** shows the sequence of the stop-jump from landing through take-off.

Test protocols are shown in Figure 3. Participants performed 3 SL tests followed by 3 SJ tests, with 30 s of rest between attempts and 1 min of rest between maneuvers (Martin et al., 2015). For the SL test, participants were asked to perform a natural jump from a 40-cm high box located 20 cm away from the force plates. The height of the drop was chosen to increase the impact forces to make it easier to detect MF while maintaining a safe landing. After landing on the preferred stance leg, participants were asked to stabilize and hold the position for at least 3 s without any secondary movement (stable and continuous) (Xu et al., 2024). For the SJ test, participants ran from 5–10 m and landed on the force platforms simultaneously with both feet for a stop jump, then immediately performed a maximal vertical jump (full power, maximal height). The movement had to be continuous without visible deviation (Milner et al., 2011; Peebles et al., 2021). Between each test, participants returned immediately to the start position and rested quietly without engaging in any additional activities or conversation, with only essential instructions provided by experimenters and no verbal encouragement or feedback given.

#### 2.4.3 Measured variables

Building on the established NC-ACLI mechanism and the critical role of multiplanar biomechanics variables(Boden and Sheehan, 2022; Beaulieu et al., 2023; Chou et al., 2023), we measured several key variables. Kinematic assessments included the three-dimensional knee angles (sagittal, frontal, and horizontal) of the preferred stance leg at peak vGRF, along with hip and ankle flexion angles in the sagittal plane. Kinetic parameters comprised the peak vGRF and the corresponding three-dimensional knee moments. Electromyographic analysis captured SEMG signals from the rectus femoris, biceps femoris (long head), tibialis anterior, and gastrocnemius lateral head during the landing cushioning phase, from which median frequency (MDF) and root mean square (RMS) were extracted. These variables have been validated as reliable and sensitive markers of NC-ACLI-related biomechanical alterations in the lower limb (Koga et al., 2018; Boden and Sheehan, 2022; Chou et al., 2023; He et al., 2024).

### 2.5 Data processing

The raw 3D marker coordinates and ground reaction data were preprocessed using a fourth-order zero-phase Butterworth low-pass filter with cutoff frequencies of 10 Hz (Winter 2009) and 50 Hz (Teng et al., 2017), to remove high-frequency noise and retain effective motion signals (Kristianslund et al., 2012). Joint centers were calculated as follows: hip centers were the midpoints of the anterior and posterior superior iliac spines; knee centers, the midpoints of the medial and lateral femoral condyles; and ankle centers, the midpoints of the medial and lateral ankles. Joint angles were calculated using the Euler method and defined as hip angles (thigh relative to pelvis), knee angles (calf relative to thigh), and ankle angles (foot relative to calf) (Wu et al., 2002). Net joint moments were computed by inverse dynamics and normalized to body weight (Robertson et al., 2013). Positive and negative values were defined as follows: sagittal plane—flexion (ankle dorsiflexion) positive, extension (ankle plantarflexion) negative; frontal plane—adduction positive, abduction negative; horizontal plane—internal rotation positive, external rotation negative.

Raw SEMG signals were bandpass filtered at 20–500 Hz using MR3.16 (Noraxon United States Inc., Scottsdale, AZ, United States) software (Luca, 1997) and segmented into phases. The cushioning phase was defined as the period from the first contact of the preferred stance leg with the force platform, where vGRF exceeded 10 N, to the maximum knee flexion angle (Cortes et al., 2012). Preprocessed SEMG data were imported into MATLAB R2023a (MathWorks, Natick, MA, United States) for further analysis, including MDF computation via Fast Fourier Transform (Luca, 1997). Filtered signals were rectified and smoothed with a 10 Hz low-pass filter to extract the envelope for RMS calculation (Hermens et al., 1999). To minimize inter-individual variability, RMS values were normalized to each subject’s maximum RMS across all trials (Besomi et al., 2020; Lanza et al., 2023)。

### 2.6 Statistical analysis

For all analyses in this study, the kinematic, kinetic, and SEMG indicators were averaged across three tests for each maneuver. Prior to statistical analysis, the normality of each item was first evaluated using the Shapiro-Wilk test and Q-Q plots, specifically including the VAS-MF scores of the pairwise differences between the three time points (pre-MF, post-MF, final-MF) (post-pre, final-pre, post-final), as well as the kinematic, kinetic, and SEMG data measured under the conditions of the pre-MF and post-MF time points and the SJ and SL maneuvers. If the difference or measurement distribution was normal, one-way repeated measures ANOVA was used for the VAS-MF scores, with partial eta-squared ( $\eta_{p}^{2}$ ) reported; for the kinematic, kinetic, and SEMG data, a two-way repeated measures ANOVA was applied, and $\eta_{p}^{2}$ was also reported. If the Mauchly sphericity assumption was violated, the Greenhouse-Geisser correction was used. For any significant interaction effects, *post hoc* comparisons were conducted via the Bonferroni method, with *P* < 0.05 considered statistically significant after adjusting for multiple comparisons. If the data still significantly deviated from normality after transformation, the Friedman test was used for the VAS-MF scores (with significant pairs compared *post hoc* and Kendall’s *W* reported as the effect size), while for the kinematic, kinetic, and SEMG data, the rank-based nonparametric repeated measures method nparLD (ANOVA-type statistic) was adopted (Worts et al., 2022), as the original settings of the nparLD method limit its results to not include effect sizes (Noguchi et al., 2012). Data that conformed to normal distribution were presented as mean ± standard deviation (M ± SD), while data that deviated from normality were expressed as median and interquartile range [M (IQR)]. All tests were two-sided, and a significance level of α = 0.05 was uniformly adopted for both overall and *post hoc* analyses. Statistical analyses were conducted in SPSS 23.0 (IBM Corp., Armonk, NY, United States) and RStudio 2024.9.0.0.0 (Posit Software, Boston, MA, United States), employing the R packages nparLD, readxl, writexl, coin, and psych. The combination of parametric and nonparametric methods ensures the robustness and reliability of the results.

## 3 Results

### 3.1 Participant characteristics and VAS-MF scores

Despite stringent quality control, data from three participants were excluded due to marker detachment, leaving 36 for analysis. The Friedman test showed significant differences among the three VAS-MF measurement time points (*X* ^2^ = 45.296, *W* = 0.62, *P* < 0.001). Pairwise comparisons revealed significant increases from pre-test to post-test (*P* < 0.001) and from pre-test to final-test (*P* < 0.001), whereas no significant difference was found between post-test and final-test (*P* = 0.472). Baseline characteristics and MF induction results are summarized in Table 2.

**TABLE 2:** Descriptive characteristics and vas-MF scores pre- and Post-MF induction.

Characteristics	Descriptive statistics (n = 36)
Age, years, M ± SD	22.1 ± 2.2
Height, cm, M ± SD	180.0 ± 60
Body mass, kg, M ± SD	76.7 ± 8.4
Body mass index, kg/m^2^, M ± SD	23.7 ± 2.0
Physical activity, times/week, M ± SD	5.0 ± 1.1
Pre-MF score, M(IRQ)	24.5 (20.0)
Post-MF score, M(IRQ)	54.0 (39.5)
Final-MF score, M(IRQ)	51.0 (37.8)
**Δ**VAS-MF score (Post - Pre), M(IRQ)	20.0 (22.5)
**Δ**VAS-MF score (Final - Pre), M(IRQ)	16.5 (23.8)
**Δ**VAS-MF score (Post - Final), M(IRQ)	4.0 (12.0)

### 3.2 Kinematics

At the moment of peak vGRF (Table 3), ANOVA revealed a significant interaction between MF status and maneuver type for the knee flexion angle (*P* = 0.019, $\eta_{p}^{2}$ = 0.148). Simple effects analysis showed that the knee flexion angle in SJ was significantly greater pre-MF than post-MF (*P* = 0.006), while the difference for SL was not significant (*P* = 0.076). The knee flexion angle in SL was consistently smaller than in SJ, both pre-MF and post-MF (*P* < 0.001 for both). For the knee internal rotation angle, a significant interaction between MF status and maneuver type was found (*P* = 0.015, $\eta_{p}^{2}$ = 0.158). Simple effects analysis indicated that the knee internal rotation angle in SJ was significantly greater pre-MF than post-MF (*P* = 0.003), whereas no significant difference was observed for SL (*P* = 0.444). Pre-MF, the knee internal rotation angle in SJ was significantly greater than in SL (*P* = 0.003), but this difference was not significant post-MF (*P* = 0.631). No significant interactions were observed between MF status and maneuver type in the ankle dorsiflexion angle (*P* = 0.570, $\eta_{p}^{2}$ = 0.099), hip flexion angle (*P* = 0.588, $\eta_{p}^{2}$ = 0.008), or knee adduction angle (*P* = 0.601, $\eta_{p}^{2}$ = 0.008). Main effects analysis showed that the ankle dorsiflexion angle was significantly smaller post-MF than pre-MF (*P* = 0.002, $\eta_{p}^{2}$ = 0.237), while the hip flexion angle (*P* = 0.891, $\eta_{p}^{2}$ = 0.001) and knee adduction angle (*P* = 0.287, $\eta_{p}^{2}$ = 0.032) showed no significant differences between pre-MF and post-MF. Regardless of MF status, the knee adduction angle (*P* = 0.004, $\eta_{p}^{2}$ = 0.216) and hip flexion angle (*P* < 0.001, $\eta_{p}^{2}$ = 0.922) were consistently smaller in SL than in SJ, while the ankle dorsiflexion angle showed no significant difference between the two movements (*P* = 0.720, $\eta_{p}^{2}$ = 0.004).

**TABLE 3:** Comparative Analysis of Kinematics and Kinetics Parameters Across Maneuver Types and Mental Fatigue status.

Parameter	Stop-jump		Single-leg landing		MF		Maneuver		MF × maneuver	
	Pre-MF	Post-MF	Pre-MF	Post-MF	*F*( $\eta_{p}^{2}$ )	*P*	*F*( $\eta_{p}^{2}$ )	*P*	*F*( $\eta_{p}^{2}$ )	*P*
Ankle dorsiflexion angle, deg, M ± SD	8.1 ± 11.7	2.7 ± 11.3	6.5 ± 3.9	5.3 ± 3.6	10.866 (0.237)	0.002	0.130 (0.004)	0.720	3.865 (0.099)	0.057
Hip flexion angle, deg, M ± SD	44.6 ± 11.1	44.0 ± 11.0	17.4 ± 7.3	17.6 ± 7.3	0.019 (0.001)	0.891	414.597 (0.922)	<0.001	0.299 (0.008)	0.588
Knee flexion angle, deg, M ± SD	55.6 ± 18.5	46.0 ± 18.1	27.4 ± 7.6	25.9 ± 7.2	9.864 (0.220)	0.003	88.174 (0.716)	<0.001	6.088 (0.148)	0.019
Knee adduction angle, deg, M ± SD	7.7 ± 15.9	9.8 ± 12.8	4.4 ± 8.9	5.7 ± 8.0	1.169 (0.032)	0.287	9.661 (0.216)	0.004	0.279 (0.008)	0.601
Knee internal rotation angle, deg, M ± SD	8.1 ± 11.7	2.5 ± 11.4	2.8 ± 10.5	1.8 ± 10.6	6.528 (0.157)	0.015	5.542 (0.137)	0.024	6.572 (0.158)	0.015
Peak vertical ground reaction force, N·kg^-1^, M (IRQ)	17.21 (5.00)	19.20 (6.85)	58.53 (12.95)	60.76 (14.32)	10.549	0.001	283.263	<0.001	1.230	0.267
Knee flexion/extension moment, Nm·kg^-1^, M ± SD	0.51 ± 1.16	−0.17 ± 1.13	0.98 ± 1.50	0.45 ± 1.66	11.372 (0.245)	0.002	5.318 (0.132)	0.027	0.311 (0.009)	0.581
Knee adduction/abduction moment, Nm·kg^-1^, M ± SD	0.21 ± 0.61	−0.24 ± 0.53	0.93 ± 0.79	0.97 ± 0.82	8.861 (0.202)	0.005	37.989 (0.520)	<0.001	5.664 (0.139)	0.023
Knee internal/external rotation moment, Nm·kg^-1^, M (IRQ)	0.09 (0.19)	0.03 (0.07)	0.25 (0.18)	0.26 (0.22)	12.159	<0.001	139.811	<0.001	8.607	0.003

Note: MF, mental fatigue; M ± SD, represents the mean and standard deviation for normally distributed data, analyzed using two-factor repeated measures ANOVA; M (IQR) represents the median and interquartile range for non-normally distributed data, analyzed using rank-based non-parametric repeated measures ANOVA.

### 3.3 Kinetics

ANOVA results (Table 3) showed a significant interaction between MF status and maneuver type for knee adduction/abduction moments (*P* = 0.023, $\eta_{p}^{2}$ = 0.139). Simple effects analysis indicated that in SJ, the knee adduction moment was significantly smaller post-MF than pre-MF (P < 0.001), while the knee abduction moment was significantly larger post-MF (P < 0.001). In SL, no significant changes were observed (*P* = 0.793). Pre-MF, the knee adduction moment of SL was significantly larger than SJ (*P* = 0.001), but post-MF, it was significantly smaller (*P* < 0.001). For knee flexion/extension moments, no significant interaction was found (*P* = 0.581, $\eta_{p}^{2}$ = 0.009), but main effects analysis revealed that knee flexion moments were significantly smaller post-MF than pre-MF for both movements (*P* = 0.002, $\eta_{p}^{2}$ = 0.245), and SL had significantly larger flexion moments than SJ (*P* = 0.027, $\eta_{p}^{2}$ = 0.132). nparLD analysis showed a significant interaction between MF status and maneuver type for knee internal/external rotation moments (*P* = 0.003). The knee internal rotation moment in SJ was significantly smaller post-MF than pre-MF (*P* = 0.001), while no significant changes were observed in SL (*P* = 0.974). For both pre-MF and post-MF, the knee internal rotation moment in SJ was significantly larger than SL (*P* < 0.001 for both). For peak vGRF, no significant interaction was found (*P* = 0.267), but main effects analysis showed that GRF was significantly greater post-MF than pre-MF for both movements (*P* = 0.001), and SL had significantly larger GRF than SJ (*P* < 0.001).

### 3.4 Electromyography

For SEMG MDF, ANOVA results (Table 4) indicated a significant interaction between MF status and maneuver type for biceps femoris MDF (*P* < 0.001, $\eta_{p}^{2}$ = 0.489). Simple effects analysis revealed that in SL, the biceps femoris MDF was significantly lower post-MF than pre-MF (*P* < 0.001), while no significant change was observed in SJ (*P* = 0.114). Pre-MF, there was no significant difference in biceps femoris MDF between SL and SJ (*P* = 0.154), but post-MF, the MDF in SL was significantly lower than SJ (*P* < 0.001). For tibialis anterior MDF, no significant interaction was found (*P* = 0.052, $\eta_{p}^{2}$ = 0.104) and the main effect of MF status was not significant (*P* = 0.059, $\eta_{p}^{2}$ = 0.098). However, the main effect of movement showed that SJ had significantly higher tibialis anterior MDF than SL both pre-MF and post-MF (*P* < 0.001, $\eta_{p}^{2}$ = 0.437). nparLD analysis revealed no significant interaction between MF status and maneuver type for the rectus femoris MDF (*P* = 0.118) or the lateral gastrocnemius MDF (*P* = 0.692). The main effect of MF status was also not significant (rectus femoris: *P* = 0.636; lateral gastrocnemius: *P* = 0.245). However, the main effect of movement showed that SJ had significantly higher lateral gastrocnemius MDF than SL (*P* < 0.001), while rectus femoris MDF was lower in SJ than SL (*P* = 0.011).

**TABLE 4:** Comparative Analysis of SEMG Parameters Across Maneuver Types and Mental Fatigue status.

Parameter	Stop-jump		Single-leg landing		MF		Maneuver		MF × maneuver	
	Pre-MF	Post-MF	Pre-MF	Post-MF	*F*( $\eta_{p}^{2}$ )	*P*	*F*( $\eta_{p}^{2}$ )	*P*	*F*( $\eta_{p}^{2}$ )	*P*
MDF, Hz										
*Rectus femoris, M (IRQ)*	44.57 (9.45)	45.45 (12.28)	47.66 (12.10)	46.65 (9.61)	0.224	0.636	6.434	0.011	2.438	0.118
*Biceps femoris, M ± SD*	35.80 ± 7.95	37.79 ± 11.19	38.45 ± 13.26	29.42 ± 10.93	12.772 (0.267)	0.001	3.701 (0.096)	0.063	33.554 (0.489)	<0.001
*Tibialis anterior, M ± SD*	107.08 ± 25.11	87.11 ± 25.92	92.97 ± 22.90	86.36 ± 22.22	3.798 (0.098)	0.059	27.164 (0.437)	<0.001	4.058 (0.104)	0.052
*Lateral gastrocnemius, M (IRQ)*	50.21 (16.92)	62.03 (21.47)	45.57 (37.67)	57.86 (22.01)	1.352	0.245	14.673	<0.001	0.157	0.692
RMS										
*Rectus femoris, M (IRQ)*	0.42 (0.06)	0.46 (0.08)	0.41 (0.06)	0.41 (0.07)	0.351	0.553	19.347	<0.001	4.098	0.043
*Biceps femoris, M (IRQ)*	0.38 (0.07)	0.43 (0.08)	0.38 (0.06)	0.38 (0.05)	1.965	0.161	10.629	0.001	2.879	0.090
*Tibialis anterior, M (IRQ)*	0.36 (0.05)	0.37 (0.07)	0.35 (0.08)	0.35 (0.06)	0.899	0.343	3.692	0.055	0.720	0.396
*Lateral gastrocnemius, M ± SD*	0.36 ± 0.04	0.37 ± 0.03	0.35 ± 0.04	0.35 ± 0.04	1.463 (0.040)	0.235	5.483 (0.135)	0.025	1.020 (0.028)	0.319

Note: MF, mental fatigue; M ± SD, represents the mean and standard deviation for normally distributed data, analyzed using two-factor repeated measures ANOVA; M (IQR) represents the median and interquartile range for non-normally distributed data, analyzed using rank-based non-parametric repeated measures ANOVA.

For SEMG RMS, nparLD analysis showed a significant interaction between MF status and maneuver type for rectus femoris RMS (*P* = 0.043). No significant changes in RMS were observed pre-MF or post-MF in SJ (*P* = 0.119) or SL (*P* = 0.349). However, post-MF, the difference in rectus femoris RMS between the two movements became significant (*P* < 0.001) after being non-significant pre-MF (*P* = 0.091). Furthermore, no significant interactions or main effects of MF status were found for the RMS of the biceps femoris (*P* = 0.090), tibialis anterior (*P* = 0.396), or lateral gastrocnemius (*P* = 0.319, $\eta_{p}^{2}$ = 0.028). However, the main effect of movement showed that RMS was consistently lower in SL than SJ for the biceps femoris (*P* = 0.001), tibialis anterior (*P* = 0.055), and lateral gastrocnemius (*P* = 0.025, $\eta_{p}^{2}$ = 0.135) regardless of MF status.

## 4 Discussion

This study aimed to examine and compare the effects of MF on biomechanical characteristics related to NC-ACLI in male college students during SJ and SL maneuvers, evaluate its potential impact on NC-ACLI risk, and to provide a preliminary discussion of possible underlying mechanisms based on the observed results and relevant literature. The results showed that MF significantly reduced ankle dorsiflexion angle and knee flexion moment and increased peak vGRF in both maneuvers, with no significant changes in hip flexion angle or SEMG RMS across the four tested muscles. Additionally, in SJ, MF significantly reduced knee flexion angle, knee internal rotation angle, and knee internal rotation moment, while increasing knee abduction moment, whereas no significant changes were observed in SL. For SEMG MDF, MF significantly decreased the biceps femoris MDF in SL, with no corresponding change in SJ. These findings support the hypothesis that MF affects NC-ACLI-related biomechanical characteristics and risk, particularly in SJ, but the precise mechanisms require further investigation.

### 4.1 Interpretation of results

The present findings suggest that, in the post-MF condition, the ACL may face greater axial loading due to increased vGRF and reduced knee and ankle flexion angles in the sagittal plane, thereby elevating NC-ACLI risk—particularly in the SJ. Specifically, in the post-MF condition, both maneuvers showed a significant rise in peak vGRF, implying increased axial compressive forces on the knee at landing. Previous work indicates that ACL loading gradually intensifies from initial foot contact and peaks when vGRF is maximal (Cerulli et al., 2003). For a 70 kg athlete, peak vGRF can range from 2 to 18 times body weight (McNitt-Gray, 1993). If these impact forces are not adequately attenuated, ACL loading may surpass its injury threshold (Woo et al., 1991), ultimately leading to NC-ACLI. Consequently, actively increasing hip, ankle, and knee flexion angles and moments is regarded as an effective cushioning strategy (Boden et al., 2010; Markolf et al., 2018; Bates et al., 2019). However, under post-MF, our study observed no significant change in hip flexion angles for either maneuver, whereas ankle dorsiflexion angle and knee flexion moment decreased significantly, with the SJ also exhibiting a pronounced reduction in knee flexion angle. This indicates a more upright or “locked” landing posture, leading to higher stiffness and diminished muscular energy absorption, which may markedly increase ACL loading, thus increasing the risk of NC-ACLI.

Moreover, in the knee’s frontal and horizontal planes, the SJ maneuver showed a significant decrease in knee internal rotation angle and moment, as well as a significant increase in knee abduction moment, whereas the SL maneuver displayed no notable kinematic or kinetic changes. From the perspective of the ACL’s multiplanar injury mechanisms, reduced internal rotation angle and moment may reflect a compensatory response (Bates et al., 2017) or point to restricted knee rotation and diminished multiplanar control, culminating in “destabilization” at landing (Beaulieu et al., 2023). Meanwhile, heightened knee abduction moment indicates that the ACL is subjected to not only increased axial compression but also greater shear and abduction forces (Kiapour et al., 2016). Such multiplanar loading may further lower the ACL’s injury threshold, substantially increasing injury risk under post-MF. By contrast, in SL maneuvers, only axial loading and reduced joint cushioning are observed, without additional shear or torsional stresses. Therefore, in the post-MF condition, SJ maneuvers may more readily reach the multiplanar risk threshold for ACL injury, significantly elevating the likelihood of NC-ACLI.

### 4.2 Potential mechanisms

MF may impair central nervous system (CNS) function, causing proprioceptive deficits and reaction time delays (7%–12%) (Van Cutsem et al., 2019; Peters et al., 2022), which disrupt neuromuscular activation and motor unit recruitment in lower limb muscles (Morris and Christie, 2020). During landing cushioning, increased gastrocnemius strength effectively absorbs GRF (Morgan et al., 2014). Combined with enhanced hamstring and soleus strength, this generates greater posterior tibial shear, reducing ACL loading (Maniar et al., 2022a). However, in this study, no significant changes in SEMG RMS were observed for the rectus femoris, biceps femoris, tibialis anterior, or lateral gastrocnemius during landing cushioning post-MF induction, suggesting no increase in muscle activation levels. This contrasts with Pageaux et al. (2015) findings in cycling, where MF increased RMS in the lateral femoral muscles. Van Cutsem et al. (2017) suggested that MF may alter motor control strategies, requiring higher central motor commands and muscle recruitment (reflected as increased SEMG amplitude) to maintain power output without exacerbating fatigue. The lack of an increase in RMS in this study may be due to differences in maneuver type and demands. While (Pageaux et al., 2013) examined sustained, rhythmic cycling, this study focused on rapid, single-execution SJ and SL maneuvers. In these relatively complex and high-speed actions, an MF-affected CNS may fail to regulate neuromuscular responses promptly, preventing a rapid rise in muscle activation levels. By contrast, during repetitive maneuvers, feedback mechanisms might be activated, increasing muscle recruitment to compensate for performance declines (Cheung and Seki, 2021). MF’s impact on the neuromuscular system may be more pronounced during short, explosive movements, leading to changes in motor unit recruitment frequency or patterns and affecting muscle function.

In this study, SEMG MDF of the biceps femoris significantly decreased in SL, while no similar change was observed in SJ. A decrease in MDF is commonly regarded as a marker of muscle fatigue, indicating reduced muscle fiber conduction velocity and altered motor unit recruitment patterns (Luca, 1997). The distinct outcomes between SL and SJ may be attributed to their differing biomechanical demands. SL emphasizes single-leg balance and stability, with the biceps femoris playing a critical role in maintaining knee joint stability and controlling flexion-extension (Chaubet and Paillard, 2012). Under MF, participants likely relied more heavily on the biceps femoris for balance, increasing its load and resulting in reduced MDF. In contrast, SJ, involving bilateral landings, distributed the load across both legs, reducing the relative burden on the biceps femoris and preventing significant changes in MDF.

Furthermore, MF may also adversely affect the feedforward control mechanisms of the landing. Feedforward control allows the CNS to anticipate task demands and proactively stabilize joints and activate muscles (Takakusaki, 2017), thereby mitigating injury risk. Kong et al. (2023) noted that patients with functional ankle instability showed higher ankle loading rates on the injured side during side-step cutting maneuvers under MF—a finding that implies compromised feedforward control, given that these patients primarily rely on feedforward mechanisms for postural adjustment (Delahunt et al., 2006). Therefore, in this study, MF may likewise have caused insufficient anticipatory muscle activation and compromised feedforward regulation—particularly in SJ maneuver demanding rapid, precise control—thereby altering biomechanical characteristics and elevating NC-ACLI risk.

Differences in the effects of MF on biomechanical metrics between SJ and SL maneuvers may be attributed to limited CNS resources, altered cognitive performance, and attention allocation (Lew and Qu, 2014; Noé et al., 2021). MF may lead to glutamate accumulation in the lateral prefrontal cortex, disrupting synaptic transmission and impairing cognitive processes, resulting in reduced cognitive performance (Proost et al., 2022; Wiehler et al., 2022). This cognitive decline can weaken attention allocation or reduce its efficiency, thereby hindering the ability to perform complex, finely controlled maneuvers while simultaneously achieving external goals (dual-tasking or multitasking) (Jiménez-Martínez et al., 2024), especially in tasks that require focusing attention on specific goals rather than the maneuvers themselves. In the relatively complex SJ maneuver, participants may focus on the goal of “maximal-height,” which could reduce their control over the maneuver and potentially lead to significant changes in biomechanical characteristics. In contrast, the simpler SL maneuver may allow participants to concentrate more on the maneuver itself to “maintain balance quickly,” thereby preserving their original maneuver patterns to a greater extent (Chaubet and Paillard, 2012; Wang et al., 2024). Consequently, the impact of MF is more pronounced in SJ. This finding is supported by other studies. Smith et al. (2016a) and Badin et al. (2016) reported that MF significantly impaired key skills like passing and shooting in soccer players, aligning with this study’s findings. Kong et al. (2023) found that MF, more than muscle fatigue, was more likely to alter ankle biomechanics in patients with functional ankle instability during unintended lateral cuts. On the other hand, previous studies have shown that MF has no significant effect on maximal strength, explosive power, or anaerobic exercise capacity—abilities that rely on peripheral muscle properties and energy metabolism rather than maneuver techniques (Pageaux et al., 2013; Duncan et al., 2015; Pageaux et al., 2015). This further highlights that MF primarily impacts tasks requiring complex cognitive and neural control, with minimal influence on tasks dependent on peripheral muscle characteristics and energy metabolism.

## 5 Strengths and practical implications

This study addresses a longstanding gap in ACL injury risk research by empirically examining the independent role of MF and its differential impacts across distinct movement tasks. Through innovative investigation of the task-dependent effects of MF on two high-risk landing maneuvers (SJ and SL), we preliminarily discuss how movement complexity and cognitive resource demands can modulate NC-ACLI-related biomechanical characteristics and risk under MF. These findings not only refine existing theoretical models for ACL injury risk assessment but also provide clear experimental evidence and theoretical support for understanding the influence of psychological and psychiatric factors in sports injuries. Based on these insights, we propose the following recommendations for sports training and injury prevention among male college students: 1) Integrate personalized cognitive–motor dual-task or multi-task regimens, along with neuromuscular and proprioceptive training, into sport-like or competitive scenarios to enhance muscle control, cognitive processing, and attention management; and 2) Actively monitor and manage MF in daily life by supporting psychological health, ensuring adequate rest, adjusting training intensity, or using supplements. Such measures can mitigate MF’s adverse impacts and reduce NC-ACLI risk (Azevedo et al., 2016).

## 6 Limitations

While this study provides important insights into the effects of MF on biomechanical risk factors for NC-ACLI, several limitations should be considered: 1) MF was evaluated only subjectively using VAS-MF, without objective neurophysiological measures (e.g., electroencephalography, functional near-infrared spectroscopy), which may introduce potential biases or placebo effects; 2) Although previous literature provides support, lack of recorded reaction time and EMG pre-activation data make the discussion on MF-induced feed-forward delay partly speculative. 3) Multimodal data such as neuroimaging, musculoskeletal modelling, or genetic markers were not integrated, limiting mechanistic insight. 4) A laboratory-based Stroop paradigm was used to induce MF, whereas on-field fatigue mechanisms may differ in nature and magnitude. 5) Participants were healthy male college students, selected for hormonal uniformity and to match prior MF studies, which limits generalizability to females and other populations. 6) All landings were fully predictable, and cutting or dual-task paradigms were excluded to isolate the effects of MF; however, this may further constrain ecological validity. 7) Although a familiarization session and 48-h intervals were implemented, potential motor learning effects cannot be completely eliminated as movement consistency metrics (e.g., coefficient of variation or intraclass correlation coefficient) were not assessed. Addressing these limitations through objective MF markers, reaction-time and EMG-onset metrics, dual-task and other unanticipated paradigms, multimodal data, and more diverse samples will strengthen future research and improve NC-ACLI prevention strategies.

## 7 Conclusion

MF significantly affects biomechanical characteristics associated with NC-ACLI, increasing the risk of NC-ACLI in male college students during SJ and SL maneuvers, with a more pronounced effect on SJ. The underlying mechanism may involve MF impairing CNS function, cognitive performance, and attention allocation, making tasks that primarily focus on external goals rather than the maneuver itself more prone to negative changes. It is recommended that MF’s potential effects be fully accounted for in NC-ACLI prevention strategies, with further research needed to clarify its specific mechanisms of action.

## Data Availability

The raw data supporting the conclusions of this article will be made available by the authors, without undue reservation.
