# Correction: Effects of mental fatigue on biomechanical characteristics and risk associated with non-contact anterior cruciate ligament injuries during landing

**DOI:** 10.3389/fbioe.2025.1641483

**Published:** 2025-07-09

**Authors:** Bosong Zheng, Zeyang Zhang, Zeyi Zhang, Youping Sun, Yao Xiao, Mengjie Li

**Affiliations:** ^1^ College of Physical Education and Health, East China Normal University, Shanghai, China; ^2^ Key Laboratory of Adolescent Health Assessment and Exercise Intervention of Ministry of Education, East China Normal University, Shanghai, China; ^3^ College of Physical Education, Chengdu Sports University, Chengdu, China

**Keywords:** mental fatigue, stop-jump, single-leg landing, non-contact anterior cruciate ligament injury, sports biomechanics

In the published article, there was an error. The time unit was incorrectly presented as “40 m” (meters) instead of the correct “40 ms” (milliseconds).

A correction has been made to **Introduction**, *paragraph 2*. This sentence previously stated:

“Early studies have demonstrated that NC-ACLI typically occurs within approximately 40 m after SL and SJ maneuvers…”

The corrected sentence appears below:

“Early studies have demonstrated that NC-ACLI typically occurs within approximately 40 ms after SL and SJ maneuvers…”

In the published article, there was an error. The phrase “potential underlying mechanisms” was not optimally concise and has been revised to “possible underlying mechanisms.”

A correction has been made to **Introduction**, *paragraph 6*. This sentence previously stated:

“Additionally, we provided a preliminary discussion of potential underlying mechanisms, drawing on existing literature to further our understanding of how psychological and psychiatric factors may contribute to NC-ACLI risk.”

The corrected sentence appears below:

“Additionally, we provided a preliminary discussion of possible underlying mechanisms, drawing on existing literature to further our understanding of how psychological and psychiatric factors may contribute to NC-ACLI risk.”

In the published article, there was an error. The anatomical location “internal ankle” was incorrectly included alongside “external ankle,” when only “external ankle” should have been specified.

A correction has been made to **2.4 Data collection**, *2.4.1 Instrumentation and setup*, paragraph 1. This sentence previously stated:

“Reflective marker dots were placed on 16 body locations (left/right anterior superior iliac spine, left/right anterior thigh, left/right lateral femoral condyles, left/right anterior tibialis, left/right posterior superior iliac spine, left/right internal/external ankle, left/right heel, and left/right ball of the foot).”

The corrected sentence appears below:

“Reflective marker dots were placed on 16 body locations (left/right anterior superior iliac spine, left/right anterior thigh, left/right lateral femoral condyles, left/right anterior tibialis, left/right posterior superior iliac spine, left/right external ankle, left/right heel, and left/right ball of the foot).”

In the published article, there was an error. The modal verb “can” was too definitive and has been revised to “may” to convey a more cautious interpretation.

A correction has been made to **4 Discussion**, *4.2 Potential mechanisms*, paragraph 2. This sentence previously stated:

“The distinct outcomes between SL and SJ can be attributed to their differing biomechanical demands.”

The corrected sentence appears below:

“The distinct outcomes between SL and SJ may be attributed to their differing biomechanical demands.”

The original version of this article has been updated.

